# Balance function in critical illness survivors and evaluation of psychometric properties of the Mini-BESTest

**DOI:** 10.1038/s41598-024-61745-5

**Published:** 2024-05-27

**Authors:** Marion Egger, Melanie Finsterhölzl, Alisa Buetikofer, Franziska Wippenbeck, Friedemann Müller, Klaus Jahn, Jeannine Bergmann

**Affiliations:** 1 Research Group, Department of Neurology, Schoen Clinic Bad Aibling, Kolbermoorer Str. 72, 83043 Bad Aibling, Germany; 2 Institute for Medical Information Processing, Biometry and Epidemiology (IBE), Faculty of Medicine, LMU Munich, Pettenkofer School of Public Health, Munich, Germany; 3 German Center for Vertigo and Balance Disorders, University Hospital Grosshadern, Ludwig-Maximilians-Universität München, Munich, Germany

**Keywords:** Neurological rehabilitation, Postural balance, Psychometric properties, Critical illness, ICUAW, Polyneuropathies, Outcomes research, Neurology, Public health, Neurological disorders

## Abstract

Critical illness survivors commonly face impairments, such as intensive care unit-acquired weakness (ICUAW) which is characterized by muscle weakness and sensory deficits. Despite these symptoms indicating potential balance deficits, systematic investigations and validated assessments are lacking. Therefore, we aimed to assess balance function using the Mini-BESTest, evaluate its psychometric properties, and identify associated variables. Balance was assessed post-ICU discharge (V1) and at discharge from inpatient neurorehabilitation (V2) in patients with ≥ 5 days of invasive ventilation. Mini-BESTest measurement characteristics were evaluated in an ambulatory subgroup. A multiple linear regression was conducted. The prospective cohort study comprised 250 patients (34% female, 62 ± 14 years, median ICU stay 55 days). Median Mini-BESTest scores improved significantly from V1 (5 (IQR 0–15)) to V2 (18.5 (10–23)) with a large effect size. Excellent inter-rater and test–retest reliabilities of the Mini-BESTest were observed (ICC = 0.981/0.950). Validity was demonstrated by a very high correlation with the Berg Balance Scale (ρ = 0.90). No floor or ceiling effects were detected. Muscle strength, cognitive function, cerebral disease, critical illness polyneuropathy/myopathy, and depression were significantly associated with balance. Despite significant improvements during the rehabilitation period, balance disorders were prevalent in critical illness survivors. Ongoing therapy is recommended. Due to its excellent psychometric properties, the Mini-BESTest is suitable for use in critical illness survivors.

**Registration:** The study was registered at the German Clinical Trials Register (DRKS00021753, date of registration: 2020-09-03).

## Introduction

A prolonged stay on intensive care unit (ICU) can lead to various complications, including impairments in physical health, cognitive function, mental health or a combination thereof^1^. These symptoms are referred to as post-intensive care syndrome (PICS) and occur in up to 80% of ICU survivors^2^. Even 1 year after ICU discharge, many patients continue to suffer from multiple symptoms^3^. A common example of physical health impairment after ICU treatment is the occurrence of a general muscle weakness which is termed ICU-acquired weakness (ICUAW). This weakness is mainly caused by dysfunction of muscles and nerves, namely critical illness polyneuropathy (CIP) and/or critical illness myopathy (CIM)^4^. The incidence of ICUAW can reach up to 80% of critically ill patients, whereby risk factors include prolonged duration of intensive care and mechanical ventilation, sepsis, multi-organ failure and immobility^4–6^. Negative outcomes accompany ICUAW, as associations with mortality, prolonged hospitalization, impaired and delayed functional recovery, and decreased health-related quality of life were shown^4,7^.

PICS can improve with time and it was recently reported that 70% of critical illness survivors with ICUAW could achieve a successful recovery^2,8^. However, intensive (neurological) rehabilitation is highly recommended to regain a good health status, but high-quality evidence about the effectiveness of rehabilitation programs (especially after ICU discharge) in patients with PICS and ICUAW is lacking^8–10^. However, physical therapy interventions were shown to be feasible, safe, and most often applied in rehabilitation^11,12^. To regain walking ability, patients with ICUAW benefited from physiotherapy interventions such as practicing walking, sit-to-stand training, and balance training^13^.

Muscular weakness is associated with decreased balance performance in older adults^14,15^. Furthermore, sensory impairment influences functional balance^16,17^. Since both motor and sensory deficits are frequent in ICUAW, balance impairments are likely present in these patients. As balance training was among the most frequently described interventions in the first two weeks of rehabilitation^13^, it is also likely that patients with ICUAW suffer from some kind of balance disorder. However, although there are some indications for impaired balance^11,18,19^, up to now there is no investigation of balance functions of critical illness survivors.

Good balance performance is crucial for independence in activities of daily living. Accordingly, leisure and social activities may be restricted by balance impairments. It was shown that community participation was affected in individuals post stroke and multiple sclerosis who displayed balance impairments^20–22^. Furthermore, balance impairments can lead to falls, which in turn increase fear of falling, which all consequently limit independence, participation and even health-related quality of life^23–25^. Hence, it is essential to tackle balance impairments. Alongside balance training, targeting factors linked to balance (such as muscle strength, cognitive function, and mental health^26–28^) may provide an opportunity for enhancing balance. However, neither balance nor associated factors were so far evaluated in ICU survivors.

The Mini-Balance Evaluation System Test (Mini-BESTest) is a balance assessment which was developed in 2010 as a short form of the BESTest^29^. To date, the assessment was mostly evaluated in patients affected by neurological diseases such as Parkinson’s disease, stroke, and multiple sclerosis, whereby good reliability, validity, and responsiveness were demonstrated^30,31^. In comparison with the widespread Berg Balance Scale, the superiority of the Mini-BESTest was shown in patients with balance disorders, Parkinson’s disease and stroke regarding reliability, ceiling effects and discriminative ability^32–34^. However, up to now, the psychometric properties of the Mini-BESTest have not been evaluated in patients after critical illness.

Although balance impairments seem to be a major aspect in the recovery of patients after critical illness, no investigations about manifestations, the development over time, or associated factors were done in this population. For careful systematic evaluation of balance function, well established and validated clinical assessment tools are needed. However, these are missing so far for individuals after critical illness. Therefore, the aims of this study were: (1) to describe balance function in critical illness survivors during neurorehabilitation, (2) to investigate independent variables associated with balance, and (3) to evaluate the psychometric properties of the Mini-BESTest in critical illness survivors.

## Methods

### Study population and setting

This analysis is a subanalysis of the single-center, prospective cohort study CINAMOPS (Critical Illness Polyneuropathy and Myopathy: Outcomes, Predictors and Longitudinal Trajectories), which is currently being conducted at the Schoen Clinic Bad Aibling, Germany^35^. The CINAMOPS study aims to gain more knowledge about critical illness survivors in terms of their progress during neurorehabilitation, their long-term outcome (physical, mental and cognitive health), the occurrence of ICUAW or CIP/CIM, predictors for the long-term outcomes, and the current therapy spectrum in neurological rehabilitation. The Schoen Clinic Bad Aibling is a centre for inpatient neurorehabilitation with a focus on critically affected patients (ICU, early neurorehabilitation); all patients with neurological deficits that need rehabilitation can be admitted. Five study visits were planned for every individual participating in the CINAMOPS study: V1 after discharge from ICU, V2 before discharge from neurorehabilitation, V3, V4, and V5 12, 18, and 24 months after disease onset.

All adult patients (≥ 18 years) with neurological deficits who were invasively ventilated on the ICU for at least 5 days were eligible for the CINAMOPS study. Patients were recruited at admission to neurorehabilitation, after discharge from the ICU. Exclusion criteria were (1) palliative care, (2) neuromuscular or neurologic diseases/syndromes causing a high-level of muscular weakness (Guillain-Barré syndrome, mysasthenia gravis, porphyria, Lambert–Eaton syndrome, amyotrophic lateral sclerosis, severe vasculitic neuropathy, cervical myelopathy, botulism; in accordance with^36^), (3) insufficient communicative abilities (German language skills or cognition) interfering with answering the questionnaires, (4) no muscular weakness (i.e. muscle strength according to the Medical Research Council (MRC) scale 5/5). A subgroup of consecutive participants (for whom the date of discharge was scheduled) was included in the evaluation of the psychometric properties of the Mini-BESTest. Exclusion criteria were (1) non-ambulatory patients (as a balance evaluation is not meaningful without any walking capacity) and (2) hemiplegia (as this is not a characteristic symptom in critical illness survivors).

During the study, patients received inpatient neurological rehabilitation (of individual length) with approximately 100 minutes of multi-disciplinary functional therapies per day, including physiotherapy, occupational, dysphagia, physical, neuropsychological and breathing therapies.

### Study visits and outcomes

The analysis includes two study visits: study visit 1 (V1) took place after discharge from ICU and therefore after admission to neurorehabilitation, study visit 2 (V2) was conducted shortly before discharge from neurorehabilitation. Generally, patients were discharged from ICU after weaning from mechanical ventilation and if their general condition was stable. Patients were discharged from neurorehabilitation after reaching sufficient functional improvement or if the patients had not improved over several weeks of rehabilitation. As the study visits were part of the CINAMOPS study, they included a variety of questionnaires and functional assessments.

The Mini-BESTest was the main outcome parameter to evaluate balance function^29^. This test evaluates dynamic balance and includes 14 items in the categories of anticipatory postural adjustments, postural responses, sensory orientation and balance during gait. The score ranges from 0 to 28 points, with higher scores indicating better balance. We used a validated German version of the Mini-BESTest^37^.

For a further evaluation of body function and walking ability, additional assessments were conducted. The functional ambulation categories (FAC; score 0–5) were used to classify walking ability^38^. Their good psychometric properties in neurological rehabilitation were shown^39^ and the assessment was also used in patients with ICUAW^36^. The Functional Reach test provides information about balance control^40^. The ability to reach forward in a standing position (or sitting if otherwise not possible) was measured in centimetres. Both arms and hands had to be extended. Assistive devices were not allowed. The score has been used before in neurological patients such as stroke^41^ and CIP/CIM patients^36^. The Timed Up and Go (TUG) is a well-established outcome measure to assess functional mobility in various populations^42^. The patient is asked to rise from a chair, walk 3 m, turn, walk back, and sit down again. The tester measures the time the patient needs to complete the task. A shorter time indicates better mobility. Good to excellent psychometric properties were shown in different neurological patient groups^43^. Muscle strength was evaluated by manual muscle testing using the scoring system of the Medical Research Council (MRC). The scale ranges from 0 to 5, with 5 indicating normal muscle strength. The following functional muscle groups were evaluated: shoulder abduction, elbow flexion, wrist extension, hip flexion, knee extension, ankle dorsal flexion^11,44^. Aggregating the MRC from all extremities yields a maximum cumulative score of 60. A sum score of < 48 was used as indicative for ICUAW^6,45,46^. Furthermore, the handgrip strength was measured twice per hand using a digital handheld dynamometer. We reported the maximum value and the normalized value, which was calculated as a percentage of the reference grip strength determined by the patient’s sex, age, and height^47^.

Patient characteristics and data about the ICU stay were collected retrospectively using the electronic medical record. Comorbidities were described by the Elixhauser Comorbidity Index^48^.

To evaluate the psychometric properties of the Mini-BESTest, an additional study visit was scheduled in a subgroup of patients shortly before or after V2 (± 2 days; patients’ conditions were assumed to be stable within this time period, as it was at the end of the rehabilitation phase). At this study visit, the Mini-BESTest was repeated by the same rater as at V2 (test–retest-reliability); a second rater observed the performance of the Mini-BESTest and scored the items independently (inter-rater-reliability). To evaluate validity, the FAC, the Functional Reach test, the MRC, and the TUG were used. Furthermore, the Berg Balance Scale was additionally carried out to evaluate validity. The Berg Balance Scale is one of the most widely used assessments to evaluate balance^49^. Its sound psychometric properties and good clinical utility were repeatedly demonstrated in various patient populations^50,51^. Out of 14 items, a score of 0–56 can be reached, with higher values indicating a better balance function. We used a validated German version of the Berg Balance Scale^52^. Floor- and ceiling effects were investigated at V2.

All tests were conducted by experienced physiotherapists trained to use the assessments.

### Statistical analysis

Categorical variables are presented as absolute values and percentages, continuous variables as mean ± standard deviation or median (quartile 1–quartile 3).

#### Description of balance function

Balance function was compared between the two study visits by the Wilcoxon signed rank test on paired samples as data were either non-parametric or did not follow normal distribution (as checked by the Shapiro–Wilk test and visually by means of QQ-plots). Effect sizes were calculated with r = z/√N. Chi-squared test and McNemar’s test were used for categorical values. Fisher’s exact test was applied in cases where more than 20% of cells had expected cell counts less than 5.

#### Multiple linear regression

We conducted a multiple linear regression analysis in order to capture factors being associated with balance function (dependent variable = Mini-BESTest at V2). Variable selection of the full model was based on previous literature and expert knowledge. Independent variables of the full model included age, MRC muscle sum score at V2, MoCA at V2, obesity, anxiety at V2, depression at V2, handgrip strength at V2 in % of reference, Elixhauser comorbidity scale, somatosensory deficits of the lower extremities at V2, duration of mechanical ventilation, diabetes, and CIP/CIM (yes = CIP, CIM or CIP/CIM; no = no CIP/CIM). Furthermore, the primary diagnoses cerebral disease (ischemic/hemorrhagic stroke; traumatic brain injury; hypoxia) and COVID-19 were included. Somatosensory function was investigated as sensation of light touch (thighs, plantar surfaces), of position sensation of the joints (ankle, knee, hip), and as vibration sensation (dorsum of the caput of os metatarsale I, internal malleolus and the tuberosity of the tibia; deficit if < 4/8 with tuning fork). A sensory deficit was apparent if any of the three categories was pathological. Anxiety and depression were measured by the Hospital Anxiety and Depression Scale^53^, cognitive function was measured by the Montreal Cognitive Assessment (MoCA)^54^. Walking ability (FAC) was not chosen as an independent variable as walking is included in several items of the Mini-BESTest and walking and balance are mutually dependent (high correlation of the FAC and the Mini-BESTest (ρ = 0.82)).

Variable selection was done according to the recommendations given by Heinze et al.^55^. We conducted a backward elimination with the Akaike information criterion (AIC, significance level 0.157) as the stopping criterion. Stability investigations of the selected model were performed according to Heinze et al.^55^. Bootstrap resampling with replacement (1000 replicates) was done for the calculation of inclusion frequencies, sampling distributions of regression coefficients, and model selection frequencies. Furthermore, the relative conditional bias (which measures the anticipated level of bias introduced by variable selection when a particular independent variable is chosen) was calculated as suggested in Heinze et al.^55^. Postestimation shrinkage factors were calculated using the R package “shrink”^56^. The goodness of fit of the model was assessed using the adjusted R^2^ statistic. The AIC was reported for model comparison. Assumptions for the multiple linear regression (linearity, homoscedasticity, multivariate normality and autocorrelation) were tested graphically for systematic violations in the selected model. For homoscedasticity, the Score Test for Non-Constant Error Variance was additionally computed. Multicollinearity was assessed by calculating variance inflation factors (VIF). The assumption of linear relationship was improved by excluding patients with a Mini-BESTest score of 0. After exclusion of the zero values and cases with missing data, the final dataset for the regression analysis included 169 cases. Accordingly, the events-per-variable (EPV_global_) was of 169/12 = 14.1.

#### Psychometric properties of the Mini-BESTest

The evaluation of the psychometric properties was done in accordance with the GRRAS^57^ and COSMIN guidelines^58,59^. We aimed to include at least 60 patients in the Mini-BEST evaluation, according to COSMIN’s rating of a good sample size^58^.

For the inter-rater and the test–retest reliability, the intraclass-correlation-coefficients (ICC) and the corresponding 95% confidence intervals were calculated. For the calculation of the ICC of the inter-rater reliability, the two-way random-effects model with type single and absolute agreement was applied^60^. For the ICC calculation of the test–retest reliability, the two-way mixed-effects model with type single and absolute agreement was applied^60^. The ICC was interpreted according to the recommendation of Koo and Li^60^.

To examine the inter-rater and test–retest reliabilities of each individual Mini-BESTest item, the quadratic (according to Fleiss–Cohen^61^) weighted kappa and the corresponding 95% confidence intervals were calculated. We used the more stringent interpretation as suggested by McHugh^62^ (0–0.20 no agreement, 0.21–0.39 minimal, 0.40–0.59 weak, 0.60–0.79 moderate, 0.80–0.90 strong and > 0.90 almost perfect agreement).

The minimal detectable change at the 95% confidence interval (MDC_95_) was computed using the formula ${MDC}_{95}=1.96\times SEM\times \sqrt{2}$^63^, whereby the standard error of measurement (SEM) value of the Mini-BESTest total score was calculated according to^64^: $SEM={SD}_{t} \times \sqrt{(1-{ICC}_{intrarater})}$.

Concurrent criterion validity was assessed by exploring the correlation between the Mini-BESTest and the Berg Balance Scale. Furthermore, construct validity (i.e. convergent validity) was assessed by exploring the correlation between the Mini-BESTest and the TUG, the Functional Reach test (standing position) and the FAC^65^. We expected high correlations as the assessments measure the same construct (except for the FAC) and as high correlations were shown before^32,64,66^. The Mini-BESTest score was correlated with the named assessments using the Spearman’s rank correlation coefficient (ρ), as data were ordinal scaled or did not follow normal distribution (controlled by Shapiro–Wilk test). Munro’s recommendations were used to interpret the correlation (no or very low: ρ = 0–0.25; low: ρ = 0.26–0.40; moderate: ρ = 0.41–0.69; high: ρ = 0.70–0.89; very high: ρ = 0.90–1.0)^67^.

Floor and ceiling effects were quantified at V2 for the Mini-BESTest (total cohort and balance evaluation group) and the Berg Balance Scale by calculating the percentage of patients with the minimum and maximum total score. A floor or ceiling effect was deemed present when more than 15% of the scores were at the minimum or maximum of the score range^63^. The skewness was calculated for a further investigation of the score distribution of the Mini-BESTest and the Berg Balance Scale.

Statistical analyses were performed using R version 4.3.2. A p-value ≤ 0.05 was considered significant. Missing data was not replaced.

### Ethics approval and consent to participate

The study was approved by the medical ethics committee of the Ludwig-Maximilians-Universität in Munich according to the Declaration of Helsinki (Project No. 20-166). Written informed consent was obtained from all participants (or their legal guardians).

## Results

### Study population

**Figure 1:**
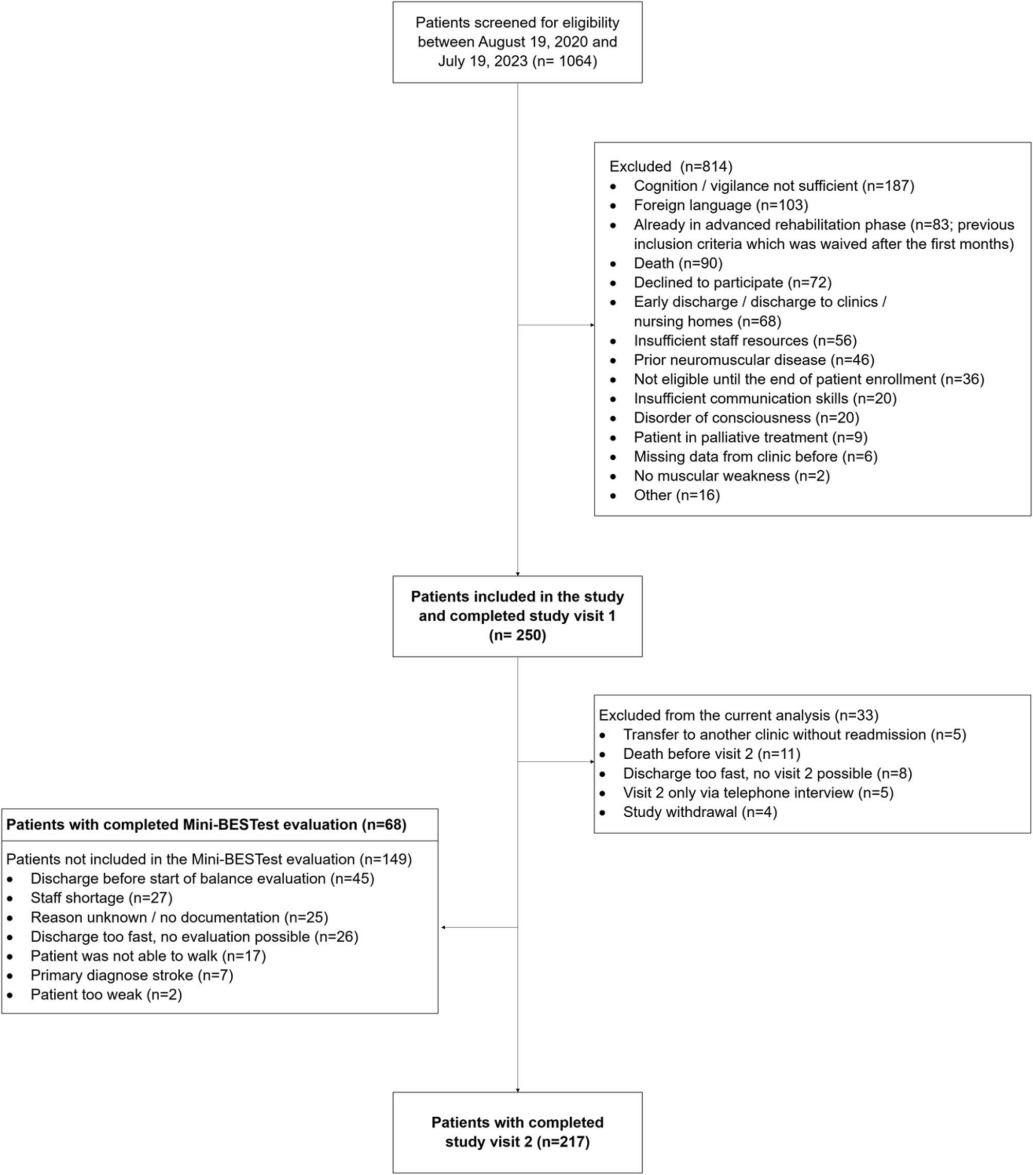
Flow chart.

A total of 1064 patients were screened between August 2020 and July 2023. 250 patients were enrolled in the study and study visits (V1 and V2) were conducted between September 2020 and December 2023 (Fig. 1). V1 was completed in 250 patients, V2 in 217 patients. 11 patients (4.4%) deceased before V2. Balance evaluations for the evaluation of the Mini-BESTest were conducted in 68 patients between September 2021 and September 2023. Patients’ characteristics are displayed in Table 1. Patients suffered frequently from acute renal failure (53.6%), sepsis (55.2%), delirium (42.4%), and acute respiratory distress syndrome (ARDS; 36.4%). According to the nerve conduction studies (performed in 216 patients), CIP, CIM or their coexistence was observed in 173 (80.1%) patients. ICUAW, as indicated by a MRC sum score < 48, was present in 216 patients (86.7%).

**Table 1:** Characteristics of included patients.

	Total group (n = 250)	Subgroup for the evaluation of psychometric properties (n = 68)
Age, years	62.4 ± 13.6, min/max: 18/92	64.9 ± 11.6, min/max: 38/88
Sex, women	86 (34.4)	20 (29.4)
Length of hospitalization, days	141 (98–193); 156.4 ± 81.8	145 (106–180); 153.2 ± 70.5
Length of ICU stay, days	55 (39–78); 64.7 ± 40.3	54 (42–73); 62.0 ± 33.3
Length of mechanical ventilation, days	39 (27–58); 45.8 ± 31.1	39 (24–57); 44.8 ± 31.8
Length of neurological rehabilitation at Schoen Clinic Bad Aibling, days	67 (44–100); 82.2 ± 60.2	63 (46–104); 80.8 ± 60.6
Time between		
First hospital admission and V1, days	81 (57–113); 90.8 ± 47.0	84 (57–111); 87.2 ± 35.9
ICU discharge and V1, days	14 (8–23); 20.3 ± 18.4	14 (7–26); 20.0 ± 18.3
V1 and V2, days	52 (29–82); 64.8 ± 50.8	48 (29–76); 59.3 ± 43.2
Primary diagnosis		
COVID-19	67 (26.8)	15 (22.0)
Cardiac disease	46 (18.4)	15 (22.0)
Pulmonary disease	45 (18.0)	15 (22.0)
Gastrointestinal/urological disease	25 (10.0)	8 (11.8)
Bacterial infection	21 (8.4)	5 (7.4)
Cerebral infarction/haemorrhage	20 (8.0)	1 (1.5)
Polytrauma	8 (3.2)	3 (4.4)
Oncological surgery	7 (2.8)	3 (4.4)
Hypoxia	5 (2.0)	2 (2.9)
Other	6 (2.4)	1 (1.5)
Nerve conduction studies^#^		
CIP	42 (19.4)	13 (21.7)
CIM	25 (11.6)	11 (18.3)
CIP/CIM	73 (33.8)	21 (35.0)
CIP but unclear CIM	22 (10.2)	3 (5.0)
No CIP but unclear CIM	11 (5.1)	2 (3.3)
No CIP/CIM	43 (19.9)	10 (16.7)
Comorbidities		
Diabetes (all type II)	47 (18.8)	15 (22.1)
Obesity	60 (24.0)	19 (27.9)
Hypertension	113 (45.2)	35 (51.5)
Elixhauser comorbidity index	4.7 ± 7.0, min/max: − 7/28	4.5 ± 6.8, min/max: − 4/27
Discharge destination*		
Home	171 (71.6)	54 (79.4)
Further rehabilitation	29 (12.1)	6 (8.8)
Home with (mobile) nursing service	18 (7.5)	5 (7.4)
Nursing home	7 (2.9)	2 (2.9)
Outpatient intensive care unit	6 (2.5)	0
Sheltered housing	4 (1.7)	1 (1.5)
Other hospital	4 (1.7)	0

Data are n (%), mean ± SD or median (quartile 1–quartile 3).

*ICU* intensive care unit, *CIP* critical illness polyneuropathy, *CIM* critical illness myopathy.

*11 patients (4.4%) died before visit 2.

^#^Electrophysiological measurement was conducted in 216 persons (86.4%). In the subgroup for the evaluation of psychometric properties, in 60 patients (88.2%) the measurement was conducted. Median time between disease onset and measurement was 91 days (67–127), the average time was 103.0 ± 50.5 days.

### Balance function

Patients’ walking and balance capabilities at V1 and V2 are displayed in Table 2. Approximately two weeks after discharge from ICU, about one third of patients was not able to walk (FAC = 0) and 44% required help for walking (FAC 1–3). Accordingly, balance function assessed using the Mini-BESTest was low at V1 (total group: 7.9 ± 8.7; subgroup with FAC > 0: 12.1 ± 8.1). Only about half of the patients were able to conduct the Functional Reach test in a standing position and the TUG. In accordance with the Mini-BESTest, the means of the Functional Reach test, and the TUG were rather low and correlated highly with the Mini-BESTest (see paragraph “Validity”).

Balance and walking improved significantly during neurorehabilitation, as shown by large effect sizes in all assessments (r = 0.547–0.831). The average Mini-BESTest score doubled to 16.3 ± 8.2 points at V2, which is reflected by a large effect size of 0.819. Nearly 75% of patients were able to walk without any help (FAC 4 and 5) at discharge from rehabilitation. Accordingly, overall muscle strength improved significantly (p < 0.001) and the majority of patients was able to do the Functional Reach test in standing position and the TUG and Dual Task TUG.

**Table 2:** Balance function and further assessments at admission to and at discharge from neurorehabilitation.

	Admission (V1)	Discharge (V2)	Test statistic	Effect size
Mini-BESTest				
Anticipatory				
1. Sit to stand	1 (0–1)	2 (1–2)		
2. Rise to toes	0 (0–1)	1 (1–2)		
3. Stand on one leg	0 (0–1)	1 (1–1)		
Reactive postural control				
4. Compensatory stepping correction—forward	0 (0–2)	2 (0–2)		
5. Compensatory stepping correction—backward	0 (0–1)	1 (0–2)		
6. Compensatory stepping correction—lateral	0 (0–1)	1 (0–1)		
Sensory orientation				
7. Stance (feet together), eyes open, firm surface	1 (0–2)	2 (2–2)		
8. Stance (feet together), eyes closed, foam surface	0 (0–1)	1 (1–2)		
9. Incline—eyes closed	0 (0–2)	2 (1–2)		
Dynamic gait				
10. Change in gait speed	0 (0–1)	2 (1–2)		
11. Walk with head turns—horizontal	0 (0–1)	1 (1–2)		
12. Walk with pivot turns	0 (0–1)	1 (0–2)		
13. Step over obstacles	0 (0–0)	1 (0–2)		
14. Timed up and go with dual task	0 (0–1)	1 (0–1)		
Mini-BESTest total score	5 (0–15); 7.9 ± 8.7	18.5 (10–23); 16.3 ± 8.3	Z = − 11.52, p < 0.001	0.819
Functional ambulation categories (FAC)				
FAC total	2 (0–3)	4 (3–5)	Z = − 11.64, p < 0.001	0.831
FAC 0: patient cannot walk	87 (34.8)	19 (8.6)		
FAC 1: patient requires physical assistance with continuous contacts	18 (7.2)	1 (0.5)		
FAC 2: patient requires physical assistance with intermittent light contact	24 (9.6)	5 (2.2)		
FAC 3: patient requires verbal supervision or stand-by help without physical contact	68 (27.2)	34 (15.3)		
FAC 4: patient can walk independently on level ground but requires help on stairs, slopes, or uneven surfaces	46 (18.4)	83 (37.4)		
FAC 5: patient can walk independently anywhere	7 (2.8)	80 (36.0)		
Functional Reach test [cm]				
Sitting position (V1 n = 115; V2 n = 29)	14.2 ± 14.0	23.2 ± 17.0	Z = − 3.15, p = 0.002	0.626
Standing position (V1 n = 232; V2 n = 211)*	10.6 ± 12.5	20.6 ± 11.4	Z = − 10.39, p < 0.001	0.751
Timed up and go (TUG) [s]				
Normal TUG (V1: n = 130, V2: 184)	15.3 (11.2–28.8) 20.3 ± 12.2	11.9 (9.0–20.2) 16.2 ± 11.4	Z = − 8.25, p < 0.001	0.796
Dual task TUG (V1: n = 114, V2: 178)	18.6 (13.4–28.5) 21.7 ± 11.5	13.9 (10.9–24.3) 19.1 ± 16.4	Z = − 7.12, p < 0.001	0.725
Muscle strength				
Muscle strength sum score (MRC)	39.1 ± 7.9	44.6 ± 7.2	Z = − 10.96, p < 0.001	0.760
Intensive care unit-acquired weakness (MRC sum score < 48)	216 (86.7)	141 (65.6)	McNemar’s χ^2^ (1) = 68.55, p < 0.001	0.245^#^
Maximum handgrip strength in kg	15.5 ± 7.5	20.5 ± 7.6	Z = − 11.20, p < 0.001	0.766
Maximum handgrip strength in % of reference	0.39 ± 0.18	0.52 ± 0.16	Z = − 11.22, p < 0.001	0.767

Data are n (%), mean ± SD or median (quartile 1-quartile 3). *n = 115 (V1)/n = 29 (V2) were only able to perform the Functional Reach test in sitting position, they were therefore assigned 0 cm in the standing position. Effect sizes are small (≥ 0.1), moderate (≥ 0.3) or large (≥ 0.5) according to Jacob Cohen: Statistical Power Analysis for the Behavioral Sciences (1988), p.79–81.

^#^ Effect size for McNemar’s test was calculated with the non-directional Cohen’s g and is interpreted as small (0.05 to < 0.15), medium (0.15 to <0.25), and large (≥0.25) according to Jacob Cohen: Statistical Power Analysis for the Behavioral Sciences (1988), p.147-149.

### Multiple linear regression

The diagnostic plots for the model assumptions (Supplementary Fig. 1) revealed slight deviations from the regression assumptions, especially regarding the normality of residuals and homoscedasticity (although the statistical test did not confirm heteroscedasticity, p = 0.116). After the backward elimination with AIC as the stopping criterion, a significant model with the independent variables MRC sum score (i.e., muscle strength), CIP/CIM, MoCA (i.e., cognitive function), cerebral disease, depression, duration of mechanical ventilation, anxiety, diabetes, and handgrip strength in % of reference was identified (Table 3). However, only muscle strength, CIP/CIM, cognitive function, cerebral disease and depression were significantly associated with balance (p < 0.032). The adjusted R^2^ for the full model was 29.5%, for the selected model 31.1%. Regarding the bootstrapping results, MRC sum score, CIP/CIM, MoCA, cerebral disease, and depression were most often selected as displayed by bootstrap inclusion frequencies > 70%. In contrast, inclusion frequencies were rather low for diabetes (50%) and even lower for grip strength (45%). Accordingly, the bootstrap median for diabetes and grip strength were 0 and therefore the model suggested by bootstrap medians differed from the selected model. Bootstrap resampling revealed the model’s instability, as it was chosen in merely 1.3% of the resamples (see Supplementary Table 1 for model frequencies). Model selection frequencies support the inclusion of the MRC sum score, CIP/CIM, MoCA, and cerebral disease, as they were always included in the ten most often selected models. In contrast, duration of mechanical ventilation, anxiety, and grip strength were only chosen in five of the ten most often selected models, which adds to the uncertainty of these variables. Accordingly, the relative conditional bias was rather high for these variables (49–70%), just like for duration of mechanical ventilation (83%). The global shrinkage factor for the selected model was 0.861. The parameterwise shrinkage factors are displayed in Supplementary Table 2.

In summary, according to the model stability investigations, the associations between balance and muscle strength (β = 0.55, shrunken β = 0.52, p < 0.001), CIP/CIM (β =  − 3.05, shrunken β =  − 2.07, p = 0.008), cognitive function (β = 0.30, shrunken β = 0.29, p = 0.015), cerebral disease (β =  − 3.48, shrunken β =  − 2.29, p = 0.018), and depression (β =  − 0.38, shrunken β =  − 0.13, p = 0.032) in patients after critical illness can be considered as confirmed. The associations between balance and duration of mechanical ventilation, anxiety, diabetes, and handgrip strength are less certain as indicated by low bootstrap inclusion frequencies, conflicting model selection frequencies, and low parameterwise shrinkage factors.

**Table 3:** Multiple linear regression analysis for the global and selected model and chosen bootstrap results.

	Global model			Selected model				Bootstrap inclusion frequency (%)	Relative conditional bias (%)	Bootstrap median	Bootstrap 95% CI
	Beta coefficient	95% CI	p-value	Beta coefficient	95% CI		p-value				
(Intercept)	− 5.69	− 18.67; 7.28	0.386	− 7.13	− 17.34; 3.09		0.170	100	1.05	− 6.03	− 18.58; 8.47
**MRC Sum Score at V2**	**0.51**	**0.32; 0.69**	** < 0.001**	**0.55**	**0.38**; **0.71**		** < 0.001**	100	0.39	0.51	0.31; 0.71
Nerve/muscle function											
No dysfunction											
** CIP/CIM**	− **2.74**	− **5.13;** − **0.35**	**0.025**	− **3.05**	− **5.31;** − **0.79**		**0.008**	83.5	10.37	− 2.68	− 4.92; 0
**MoCA at V2**	**0.31**	**0.05; 0.58**	**0.020**	**0.30**	**0.06**;** 0.54**		**0.015**	82.6	7.25	0.29	0; 0.56
**Cerebral disease**	− **3.63**	− **6.75;** − **0.52**	**0.022**	− **3.48**	− **6.36;** − **0.61**		**0.018**	82.5	13.46	− 3.57	− 6.92; 0
**Depression at V2**	− 0.35	− 0.71; 0.01	0.056	− **0.38**	− **0.72;** − **0.03**		**0.032**	74.8	25.55	− 0.35	− 0.75; 0
Duration of mechanical ventilation [days]	− 0.02	− 0.05; 0.01	0.167	− 0.02	− 0.05; 0.01		0.131	54.1	49.13	− 0.02	− 0.05; 0
Anxiety at V2	0.21	− 0.12; 0.54	0.209	0.24	− 0.08; 0.55		0.136	53.1	70.40	0.21	0; 0.61
Diabetes											
No diabetes	*Reference*										
Diabetes	− 1.44	− 3.99; 1.10	0.264	− 1.74	− 4.14; 0.66		0.153	50.1	82.96	0	− 4.42; 0
Handgrip strength % of reference	− 0.04	− 0.12; 0.03	0.233	− 0.06	− 0.12; 0.01		0.091	44.8	68.92	0	− 0.12; 0
Somatosensory function											
No impairment	*Reference*										
Somatosensory deficits	− 0.96	− 3.04; 1.13	0.365					39.1	118.17	0	− 3.39; 0
Body weight status											
No obesity	*Reference*										
Obesity	− 0.81	− 3.26; 1.64	0.514					32.3	131.60	0	− 3.24; 1.58
Age [years]	0.00	− 0.08; 0.08	0.997					26.8	14,814.57	0	− 0.10; 0.08
Elixhauser comorbidity index	− 0.04	− 0.20; 0.11	0.582					26.5	143.13	0	− 0.18; 0.10
COVID-19 disease	− 0.33	− 2.62; 1.96	0.775					21.4	148.03	0	− 2.5; 1.98
R^2^	0.334			0.347							
Adjusted R^2^	0.295			0.311							
Residual std. error	5.96 (df = 154)			5.90 (df = 159)							
F-statistic	6.02 (df = 14; 154); p < 0.001			9.41 (df = 9; 159); p < 0.001							
AIC	1099.3			1091.0							

*AIC* Akaike information criterion, *95% CI* 95% confidence interval, *MRC* Medical Research Council, *MoCA* Montreal Cognitive Assessment, *V2* Visit 2 at discharge from rehabilitation, *CIP/CIM* Critical Illness Polyneuropathy/Myopathy. The bootstrap median is zero in case a variable was chosen in < 50% of the resamples.

Significant values are in bold.

### Evaluation of the psychometric properties of the Mini-BESTest

The subgroup of patients for the Mini-BESTest evaluation was similar to the total population in all parameters (p > 0.134; Table 1, Supplementary Table 3). 39 patients (57.4%) had an MRC sum score < 48 and therefore exhibited an ICUAW. As three patients refused to repeat the Mini-BESTest on the second day, test–retest reliability could only be calculated for 65 patients. The average time between the two Mini-BESTest measurements was 1.8 ± 1.4 days. The mean Mini-BESTest score of the investigator was 18.2 ± 6.0, of the observer 18.3 ± 5.9 and of the retest was 18.1 ± 6.0. The mean score of the Berg Balance Scale was 46.1 ± 11.2.

#### Reliability

Inter-rater reliability of the Mini-BESTest total score was found to be excellent with ICC = 0.981 (95% CI 0.969–0.988), yielding an MDC_95_ value of 2.3 points with SEM = 0.87. Likewise, an excellent ICC for the test–retest reliability was found (ICC = 0.950 (95% CI 0.920–0.970), yielding an MDC_95_ value of 3.7 points with SEM = 1.34. The inter-rater reliability for the single items (Table 4) was strong or almost perfect for all items except item 11 (walk with head turns; 0.74 (95% CI 0.56–0.91)). The test–retest reliability for the single items was slightly inferior, as there were some items with only moderate agreement (items 4, 5, 6, 8; kappa 0.70–0.72); items 11 (walk with head turns) and 14 (dual task) showed only weak agreement (kappa 0.59 and 0.52).

**Table 4:** Inter-rater and test–retest reliability for the individual items of the Mini-BESTest.

	Inter-rater reliability	Test–retest reliability
Item 01—Sit to stand	0.97 (0.91–1)	0.90 (0.80–1)
Item 02—Rise to toes	0.90 (0.83–0.98)	0.82 (0.72–0.92)
Item 03—Stand on one leg	0.93 (0.84–1)	0.89 (0.77–1)
Item 04—Compensatory stepping correction—forward	0.91 (0.83–0.99)	0.72 (0.55–0.90)
Item 05—Compensatory stepping correction—backward	0.94 (0.88–0.99)	0.70 (0.56–0.85)
Item 06—Compensatory stepping correction—lateral	0.92 (0.84–0.99)	0.72 (0.59–0.85)
Item 07—Stance (feet together), eyes open, firm surface	1 (1–1)	1 (1–1)
Item 08—Stance (feet together), eyes closed, foam surface	0.87 (0.75–0.98)	0.71 (0.54–0.88)
Item 09—Incline, eyes closed	0.91 (0.83–1)	0.73 (0.58–0.88)
Item 10—Change in gait speed	0.90 (0.81–0.99)	0.88 (0.79–0.98)
Item 11—Walk with head turns—horizontal	0.74 (0.56–0.91)	0.59 (0.43–0.76)
Item 12—Walk with pivot turns	0.82 (0.72–0.92)	0.84 (0.75–0.93)
Item 13—Step over obstacles	0.90 (0.84–0.96)	0.81 (0.71–0.92)
Item 14—Timed up and go with dual task	0.93 (0.87–0.99)	0.52 (0.32–0.72)

Reliability values are measured with quadratic weighted kappa and are displayed with their 95% confidence intervals.

#### Validity

Convergent validity was demonstrated, as the correlations between the Mini-BESTest and the Berg-Balance-Scale (ρ = 0.90), the TUG (ρ =  − 0.86), the FAC (ρ = 0.82), and the Functional Reach test (ρ = 0.73) were high to very high and significant (p < 0.001).

#### Floor and ceiling effects

At V2, 212 Mini-BESTest measurements were conducted. 17 patients (8.0%) had a total Mini-BESTest score of 0, 3 patients (1.4%) scored the maximum of 28. The skewness was − 0.61. Therefore, no floor- and ceiling effects were apparent for the Mini-BESTest at discharge from rehabilitation.

**Figure 2:**
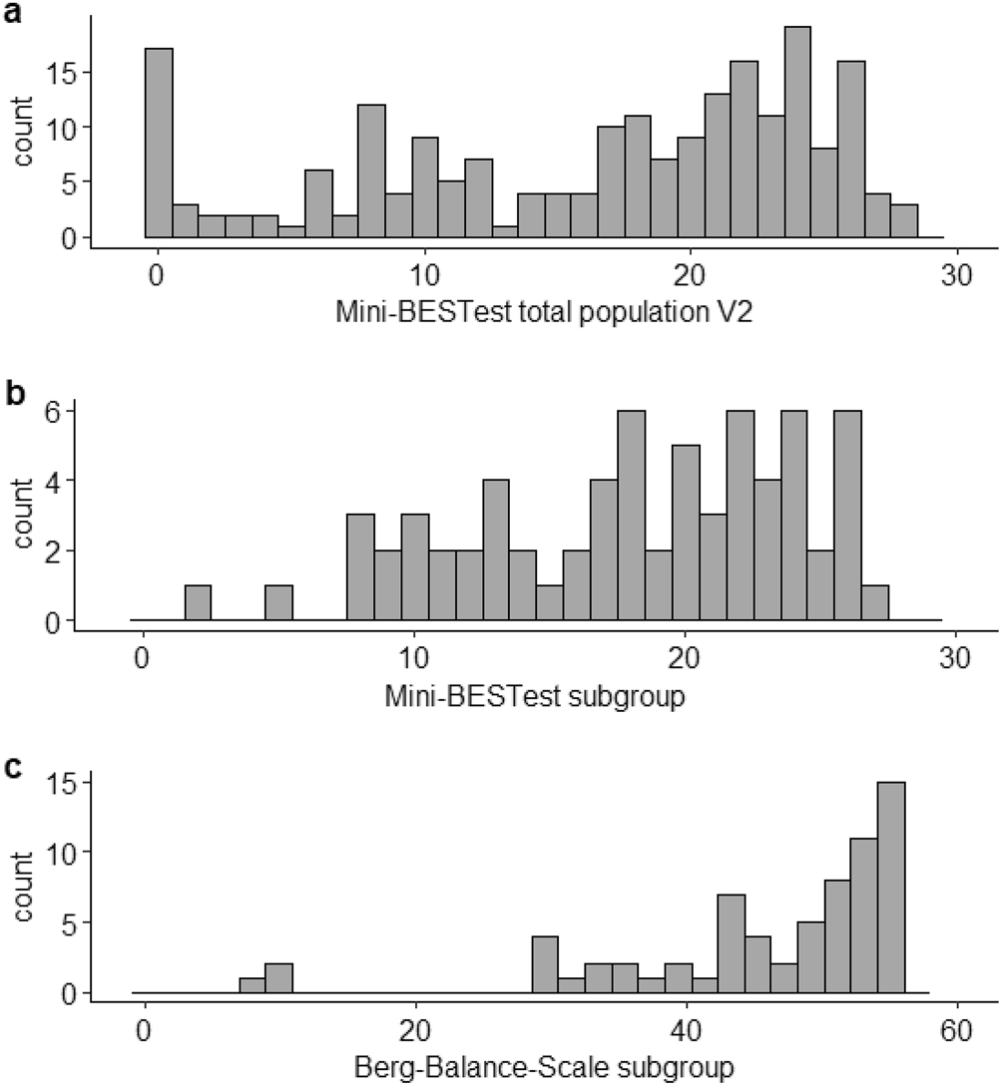
Count of total scores of the Mini-BESTest for the total population being investigated at discharge from neurorehabilitation (V2; n = 212; Figure **a**) and for the Mini-BESTest (**b**) and the Berg Balance Scale (**c**) in the subgroup of patients for the Mini-BESTest evaluation (n = 68).

Regarding the subgroup of patients for the evaluation of psychometric properties of the Mini-BESTest, no patient scored 0 on the Mini-BESTest or the Berg Balance Scale, therefore no floor effects were apparent. No patient had the maximum Mini-BESTest score (i.e., 28 points), but 10 patients had the Berg Balance Scale maximum value of 56 points (14.7%). Therefore, a trend for a ceiling effect for the Berg Balance Scale was apparent. Accordingly, the skewness of the Mini-BESTest in the subgroup was substantially lower than for the Berg Balance Scale (− 0.55 vs. − 1.71). Score distributions of the Mini-BESTest and the Berg-Balance-Scale are shown in Fig. 2.

## Discussion

### Summary of results

In this study, we aimed to describe balance function in patients after critical illness, investigate variables associated with balance and evaluate the psychometric properties of the Mini-BESTest in this group of patients. The analysis showed that the majority of the ICU survivors exhibited CIP/CIM and/or ICUAW and balance function and walking ability were highly impacted. After admission to neurorehabilitation, 35% of patients were not able to walk at all (FAC = 0) and the median Mini-BESTest score was only 5 of the maximum 28 points. Accordingly, muscle strength was substantially reduced. During neurorehabilitation, patients considerably improved balance function and walking ability. However, balance was still impaired at discharge in many participants and the median Mini-BESTest was only 18.5 of 28 points. According to the multiple linear regression, muscle strength, cognitive function, the presence of CIP/CIM, cerebral disease, and depression were associated with balance function. According to the evaluation of the psychometric properties, the Mini-BESTest was shown to be a reliable and valid tool in patients after critical illness and no floor or ceiling effects were detected.

### Mini-BESTest scores and cutoff values in other populations

Balance was rarely measured in patients after critical illness or with ICUAW before. In a population with chronic stroke patients (median stroke duration 2.9 years; mean age 57 ± 11 years), a Mini-BESTest score of 19.0 (IQR 14.0–22.0) was recorded^64^. In a sub-acute stroke population (mean stroke duration 124.4 ± 106.5 days; mean age 61 ± 9 years) admitted to an outpatient stroke rehabilitation, a mean score of 18.2 ± 6.5 points was reached^68^. In a study including individuals with Parkinson’s disease (66 ± 11 years, mostly Hoehn and Yahr stages II and III), a mean score of 18.8 ± 6.7 was reported^69^. In comparison, healthy subjects (70 ± 6 years; 60 ± 9 years) reached on average 25.3 ± 2.2 points and a median of 27 (IQR 27–28) points in the Mini-BESTest^64,70^. Based on these literature findings, balance function of ICU survivors upon discharge from rehabilitation (~ 150 days after disease onset) is comparable to that of individuals with chronic and sub-acute stroke and with Parkinson’s Disease at stages II and III, but noticeably differs from the balance function of healthy subjects of the same age group.

Cut-off values of the Mini-BESTest score were previously established to describe manifestation of balance deficits and to distinguish fallers and non-fallers in patients with Parkinson’s disease, stroke and peripheral neuropathy as well as in community-dwelling adults^33,69,71–74^. Having a cut-off for the detection of limited balance function or for fall prediction would also be of high relevance for patients after critical illness; however, this was not the subject of the current study and requires future investigation.

### Factors associated with balance

We found that muscle strength, cognitive function, the presence of CIP/CIM, the primary diagnosis cerebral disease, and depression were the key factors associated with balance. This is partly in line with previous study results, as higher muscle strength was also reported as an independent predictor for enhanced balance function in patients with chronic stroke^26^ and in older hip fracture patients after motor rehabilitation^75^. However, no such association was found in healthy individuals^76^. Cognitive function was also reported as an independent predictor for balance in patients with Parkinson’s disease^27^ and older hip fracture patients^75^. The influence of CIP/CIM on balance has not been assessed before. Patients with stroke and other cerebral diseases frequently suffer from impaired postural control and balance disorders^77–80^. Furthermore, depression was included in the selected model and the results of a review strengthen the association between depression and impaired balance^81^.

Handgrip strength was also included in the selected model with a p-value very near to the significance level. However, stability investigations indicated uncertainty about its true influence. In the literature, weaker handgrip strength was repeatedly shown to correlate with worse dynamic postural balance, e.g. in older adults and people under long-term care facilities^82–84^. Duration of mechanical ventilation was also included in the selected model; however, there was no significant p-value and stability investigations led to uncertainty regarding its association with balance. The influence of the duration of ventilation on balance had not been evaluated before. However, a longer duration of mechanical ventilation was frequently reported as risk factor for muscle weakness and CIP/CIM^46,85,86^, therefore an association is comprehensible. Diabetes was also included in the selected model, however stability investigations led to uncertainty regarding its association with balance. In contrast, previous studies supported the association between diabetes and balance impairments^87,88^. Anxiety was also included in the selected model, albeit without statistical significance and with indications of uncertainty resulting from the stability investigations. However, the influence of anxiety on postural control was frequently demonstrated, even on a neurobiological basis^89–91^. In light of the minor deviations from the regression assumptions, they might have also introduced some uncertainty into the interpretation of our results.

### Reliability and validity in comparison with previous literature

As this is the first investigation of the psychometric properties of the Mini-BESTest in patients after critical illness, no direct comparison within the same patient group is possible. However, our results align with previous investigations involving other patient groups. We found excellent values for inter-rater reliability (ICC = 0.98), which was also found in patients with balance disorders due to stroke, Parkinson’s disease and other neurological diseases (ICC = 0.86–0.99)^30^. The same applies for the test–retest reliability, where our results (ICC = 0.95) are in line with those previously reported (ICC = 0.92–0.98)^30^. For the inter-rater reliability of the single items, we received better kappa values than reported in a group of persons with chronic stroke^64^. Furthermore, in this study, values for intra-rater reliability of the single items were calculated, which can be compared to our test–retest values. They explored only fair reliability for items 5, 8, and 13, whereas we found limited reliability for items 11 and 14. The learning effect might not be causal, as the same number of participants had worse and improved values respectively on the second day of evaluation. As scheduling of the two evaluations varied (morning/afternoon after full day of therapies), this might have potentially influenced the patients’ performance and therefore also the reliability values. An MDC of 3.0^64^ and 3.5^32^ was found in patients with chronic stroke and mixed neurological diseases, which is comparable to our reported MDC of 3.7. Furthermore, validity of the Mini-BEST was confirmed and previously reported correlations with the Berg Balance Scale (r/ρ = 0.79–0.94)^30^, the TUG (r/ρ = 0.66–0.89)^30^ and the Functional Reach test (ρ = 0.55)^64^ were similar to those in our study. Neither floor nor ceiling effects were apparent in any of the previously investigated patient groups, which is in line with our results^30^. In conclusion, the Mini-BESTest appears well-suited to measuring balance in patients after critical illness.

### Clinical and scientific implications

This analysis provides evidence for the existence of balance impairments in individuals after critical illness. It was shown that a majority of patients exhibited a lack of balance control after discharge from the ICU. Accordingly, walking ability and muscle strength were substantially impaired. During neurorehabilitation, balance function (as well as walking and muscle strength) improved significantly with large effect sizes. However, due to the study design, it is uncertain to which extent the observed effects can be attributed to the rehabilitation interventions. Average balance function was still reduced at discharge from rehabilitation and the Mini-BESTest scores were comparable to individuals after stroke or with Parkinson’s disease^64,68,69^. Consequently, intensive neurorehabilitation is highly indicated for patients after discharge from ICU, especially for patients with proven CIP/CIM and cerebral diseases, and ongoing (physio-) therapy even beyond discharge from rehabilitation is recommended. As muscle strength, cognitive function, and depression were found to be significantly associated with balance, these factors should be addressed in rehabilitation therapies.

No valid clinical tool was so far available for measuring balance in patients after critical illness. According to its excellent psychometric properties, the Mini-BESTest is suitable for clinical practice and research. Regarding the almost present ceiling effect of the Berg Balance Scale at V2, the Mini-BESTest seems superior for measuring balance in patients after critical illness. Follow-up studies are required to further investigate the long-term development of balance function.

### Limitations

Conditions for the evaluation of test–retest reliability varied in some patients. Due to the full therapy schedule, balance tests were sometimes conducted at different points in the day, e.g., in extreme cases, one test was conducted in the morning whereas the retest was conducted on another day in the evening, when the patient was possibly exhausted after the rehabilitation therapies. However, this was only the case in the minority of balance evaluations and the test–retest reliability was still found to be excellent. Nevertheless, it should be acknowledged that exhaustion could influence balance function which should be considered in future studies examining ICU survivors.

Responsiveness and the minimal clinically import difference are important psychometric properties which we did not investigate in this study. Although the change in the Mini-BESTest score over the rehabilitation period was comparable to the improvements in the FAC, Functional Reach test and TUG according to the effect sizes, a comparison with the Berg Balance Scale for the responsiveness would have been more accurate and needs to be done in future studies. This also applies for the cutoff values as mentioned before.

The generalizability of our results might be limited, as our participants had extremely long durations of ICU treatment and mechanical ventilation compared to the previous studies describing the outcome of ICU survivors and patients with ICUAW^92–95^. Additionally, 26% of our cohort were participants suffering from COVID-19.

## Conclusions

Balance disorders were frequent in individuals after discharge from the ICU as well as after several weeks of neurorehabilitation. However, large effect sizes were found for the improvement of balance function over the period of rehabilitation, which suggests a potential positive effect of the therapies. Muscle strength, cognitive function, CIP/CIM, cerebral disease, and depression were significantly associated with balance function. The Mini-BESTest was shown to be a reliable and valid tool for assessing balance in individuals after critical illness and therefore seems well-suited for clinical practice and research. As balance disorders were still substantial at discharge from rehabilitation and comparable to patients with stroke and Parkinson’s disease, further follow-up investigations and therapies are required in this patient group.

### Supplementary Information

Supplementary Information.

## Data Availability

The datasets used and/or analysed during the current study are available from the corresponding author on reasonable request.
