# Supplementary material for: Balance function in critical illness survivors and evaluation of psychometric properties of the Mini-BESTest
Source: Sci Rep. 2024 May 27;14:12089. doi: 10.1038/s41598-024-61745-5 (PMC11130260; doi:10.1038/s41598-024-61745-5)
Supplement: Supplementary file 1 — Supplementary Information. [file 41598_2024_61745_MOESM1_ESM.pdf]

Supplementary material

Balance Function in Critical Illness Survivors and Evaluation of Psychometric Properties of the Mini-BESTest

Authors: Marion Egger, Melanie Finsterhölzl, Alisa Buetikofer, Franziska Wippenbeck, Friedemann Müller, Klaus Jahn, Jeannine Bergmann

Corresponding author:

Marion Egger, MSc

Mail: megger@schoen-klinik.de

Affiliations:

- Research Group, Department of Neurology, Schoen Clinic Bad Aibling, Bad Aibling, Germany
- Institute for Medical Information Processing, Biometry, and Epidemiology (IBE), Faculty of Medicine, LMU Munich, Pettenkofer School of Public Health, Munich, Germany

Supplementary Fig. 1 Diagnostic plot for model assumptions of the selected model

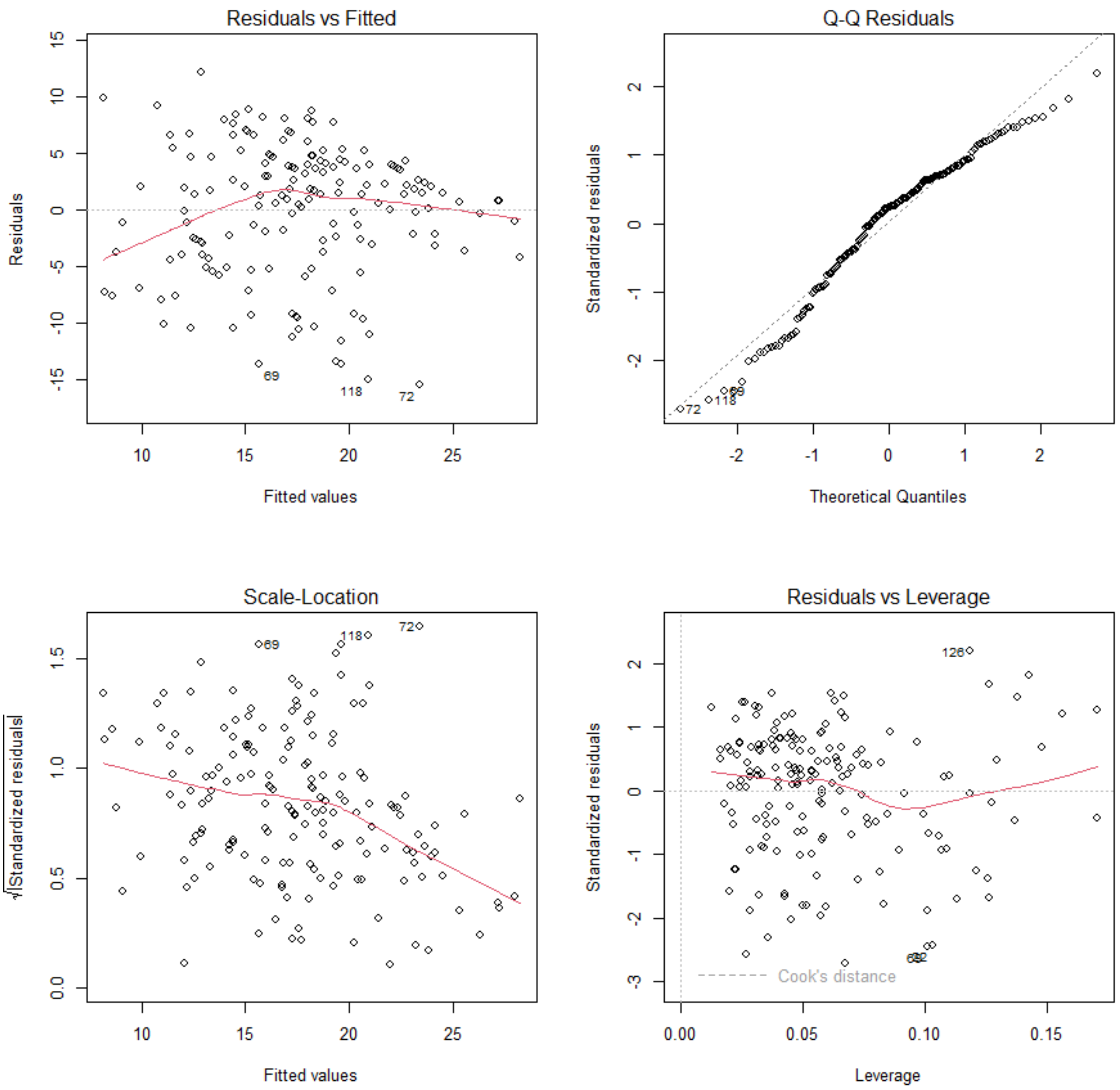

Supplementary Table 1 Model selection frequencies

Model	Included predictors	Count	Percent	Cumulative Percent
1	mrcv2 cipcim mocav2 brain depressionv2 ventilation anxietyv2 gripstrength	14	1.4	1.4
2	mrcv2 cipcim mocav2 brain depressionv2 ventilation anxietyv2 diabetes gripstrength	13	1.3	2.7
3	mrcv2 cipcim mocav2 brain depressionv2 anxietyv2 diabetes	12	1.2	3.9
4	mrcv2 cipcim mocav2 brain depressionv2 ventilation anxietyv2 diabetes	10	1	4.9
5	mrcv2 cipcim mocav2 brain depressionv2 diabetes	8	0.8	5.7
6	mrcv2 cipcim mocav2 brain depressionv2 anxietyv2	7	0.7	6.4
7	mrcv2 cipcim mocav2 brain diabetes	6	0.6	7
8	mrcv2 cipcim mocav2 brain ventilation gripstrength	6	0.6	7.6
9	mrcv2 cipcim mocav2 brain depressionv2 diabetes gripstrength	6	0.6	8.2
10	mrcv2 cipcim mocav2 brain ventilation diabetes gripstrength	6	0.6	8.8
11	mrcv2 cipcim mocav2 brain depressionv2 ventilation diabetes gripstrength	6	0.6	9.4
12	mrcv2 cipcim brain depressionv2 anxietyv2 diabetes gripstrength	6	0.6	10
13	mrcv2 cipcim mocav2 brain depressionv2 ventilation anxietyv2 gripstrength sensorydeficit	6	0.6	10.6
14	mrcv2 cipcim mocav2 brain depressionv2 ventilation anxietyv2 diabetes gripstrength	6	0.6	11.2
15	mrcv2 cipcim mocav2 brain depressionv2 ventilation gripstrength	5	0.5	11.7
16	mrcv2 cipcim mocav2 depressionv2 anxietyv2 diabetes gripstrength	5	0.5	12.2
17	mrcv2 cipcim mocav2 brain depressionv2 anxietyv2 diabetes gripstrength	5	0.5	12.7
18	mrcv2 cipcim mocav2 brain depressionv2 anxietyv2 sensorydeficit	5	0.5	13.2
19	mrcv2 cipcim mocav2 brain depressionv2 anxietyv2 diabetes sensorydeficit	5	0.5	13.7
20	mrcv2 cipcim mocav2 brain depressionv2 ventilation anxietyv2 diabetes sensorydeficit	5	0.5	14.2

Supplementary Table 2 Parameterwise shrinkage factors

	Parameterwise shrinkage factors	Selected model β-coefficients	Shrunken β-coefficients
MRC sum score	0.95	0.55	0.52
CIP/CIM	0.89	-3.05	-2.07
MoCA	0.96	0.30	0.29
Cerebral disease	0.66	-3.48	-2.29
Depression*	0.34	-0.38	-0.13
Duration of mechanical ventilation	0.59	-0.02	-0.01
Anxiety*	0.34	0.24	0.08
Diabetes	0.58	-1.74	-1.02
Handgrip strength	0.48	-0.06	-0.03

*As the parameterwise shrinkage factor for anxiety was -0.05 and correlated moderately (0.57) with the parameterwise shrinkage factor of depression (0.42), a joint shrinkage factor for anxiety and depression was calculated and numbered 0.34.

Supplementary Table 3 Characteristics of included patients and comparison of the subgroup

	Group without participants of balance evaluation (n=182)	Psychometric properties n=68	p-value
Age, years	61.4±14.1, min/max: 18/92	64.9±11.6, min/max: 38/88	0.134
Sex, women	66 (36.3)	20 (29.4)	0.387
Length of hospitalization, days	138 (97-197); 157.6±85.7	145 (106-180); 153.2±70.5	0.834
Length of ICU stay, days	55 (38-66); 65.6±42.7	54 (42-73); 62.0±33.3	0.853
Length of mechanical ventilation, days	39 (28-57); 45.9±30.9	39 (24-57); 44.8±31.8	0.722
Length of neurological rehabilitation at Schoen Clinic Bad Aibling, days	67 (42-100); 82.1±60.5	63 (46-104); 80.8±60.6	0.936
Time between…			
first hospital admission and V1	78 (58-113); 92.3±50.4	84 (57-111); 87.2±35.9	0.806
ICU discharge and V1	15 (8-23); 20.6±18.5	14 (7-26); 20.0±18.3	0.342
V1 and V2	54 (30-86); 67.4±53.8	48 (29-76); 59.3±43.2	0.297
Primary diagnosis			0.371
COVID-19	52 (20.8)	15 (22.0)	
Cardiac disease	31 (12.4)	15 (22.0)	
Pulmonary disease	30 (12.0)	15 (22.0)	
Gastrointestinal / urological disease	17 (6.8)	8 (11.8)	
Bacterial infection	16 (6.4)	5 (7.4)	
Cerebral infarction / haemorrhage	19 (7.6)	1 (1.5)	
Polytrauma	5 (2.0)	3 (4.4)	
Oncological surgery	4 (1.6)	3 (4.4)	
Hypoxia	3 (1.2)	2 (2.9)	
Other	5 (2.0)	1 (1.5)	
Nerve conduction studies[#]			0.247
CIP	29 (18.6)	13 (21.7)	
CIM	14 (9.0)	11 (18.3)	
CIP/CIM	52 (33.3)	21 (35.0)	
CIP but unclear CIM	19 (12.2)	3 (5.0)	
No CIP but unclear CIM	9 (5.8)	2 (3.3)	
No CIP/CIM	33 (18.1)	10 (16.7)	
Comorbidities			
Diabetes (all type II)	32 (17.6)	15 (22.1)	0.533
Obesity	41 (22.5)	19 (27.9)	0.468
Hypertension	78 (42.9)	35 (51.5)	0.282
Elixhauser Comorbidity Index	4.8±7.1, min/max: -7/28	4.5±6.8, min/max: -4/27	0.778

Data are n (%), mean ± SD or median (quartile 1-quartile 3)
ICU=intensive care unit, CIP=Critical Illness Polyneuropathy, CIM=Critical Illness Myopathy; [#] Electrophysiological measurement was conducted in 156 of 182 persons (85.7%). In the subgroup for the evaluation of psychometric properties, in 60 patients (88.2%) the measurement was conducted. 11 patients (4.4%) of the whole 250 patients died before visit 2. P-values were calculated with the Mann-Whitney-U test except for sex, comorbidities, and nerve conduction studies which were calculated with the Chi-squared test and the primary diagnosis, which was calculated with the Fisher´s Exact test.
